# Inter sphincter rectal resection with and without Malone ante grade continence enema in cases with low rectal cancer: A randomized, prospective, single-blind, clinical trial

**DOI:** 10.22088/cjim.13.3.546

**Published:** 2022

**Authors:** Mina Alvandipour, Mohammad Yasin Karami, Mahmood Azadfar, Jamshid Yazdani Charati

**Affiliations:** 1 Gastrointestinal Cancer Research Center, Non-Communicable Diseases Institute, Mazandaran University of Medical Science, Sari, Iran; 2 Breast Diseases Research Center, Shiraz University of Medical Sciences, Shiraz, Iran; 3 Department of Suregry, Mazandaran University of Medical Science, Sari, Iran; 4 Department of Biostatistics, Health Sciences Research Center, Faculty of Health, Mazandaran University of Medical Sciences, Sari, Iran

**Keywords:** Inter-sphincteric resection, Rectal cancer, Quality of life

## Abstract

**Background:**

Fecal incontinence is the main morbidity of inter-sphincteric resection (ISR) in ultra-low rectal cancer. Malone Ante grade Continence Enema (MACE) has been proposed for these patients. We aimed to compare the quality of life outcomes in cases with ultra-low rectal cancer who had undergone ISR±MACE.

**Methods:**

The current randomized clinical study was accomplished for two years from December 2016 to February 2018 in Imam Khomeini Hospital (Sari City, I.R.Iran) on 30 patients (15 in each group) with rectal cancer. The inclusion criteria of the study were stage 1 and 2a of low rectal cancer with type 2 and 3 of Rullier's classification, those who received neoadjuvant chemo radiotherapy. The exclusion criteria were comorbidity diseases, immune deficiency, poor follow-up. The follow-up period was one year. The Quality of Life (Qol) was reported as primary endpoint. The EORTC QLQ-C30 score and Wexner questionnaires were used. SPSS Version 22 was used. A *p*-value less than 0.05 was considered statistically significant.

**Results:**

The mean age of patients was 56.23±8.72years. The overall Qol score was better in the ISR-MACE (P=0.023). The overall Qol was lower in women than in men in both groups. Low anterior resection syndrome score was lower in the ISR plus MACE group than the ISR group (P=0.030). The Wexner score revealed better scores in the ISR with MACE group than the ISR without MACE group (p<0.0001).

**Conclusion:**

Patients who underwent ISR plus MACE surgery had better defecation control and better quality of life than patients without MACE.

Colorectal cancer has become a major medical and social problem since the beginning of the 21st century, with nearly 44,180 new rectal malignancy patients reported in the United States (1, 2). Generally, the rectal cancer survival rate has improved (3, 4). Surgical morbidity is a problem that changes the patients' lifestyle and quality of life (QoL). Almost, rectal cancer procedure requires colostomy, especially for very low rectal cancer cases who need Abdomino-Perineal Resection (APR). The presence of a colostomy is one of the most important surgical morbidities that can negatively affect the patients in the long time. Psychological complications such as anxiety about the bad smell and leakage of exhalation material or appearance of stoma from underwear that attracts the attention of others are the most important negative effects. About 25% of patients with stoma suffer from psychological disorders, including depression and anxiety, and other negative mood disorders (5).

Therefore, the use of procedures that can be addressed by patients who do not require colostomy can be useful in reducing postoperative morbidity. APR therapy is used in cases with low and ultra-low rectal cancers, and the final results are determined based on the stage of cancer and anatomical pelvic status. In cases where the external sphincter is not involved, it is possible to prevent colostomy using the Inter sphincter Resection (ISR). In recent years, ISR has been proposed as a substitute for APR in preserving the sphincter in patients with very low early-stage rectal cancers. APR is consistently related with a higher regional recurrence (greater than 22%) compared with low anterior resection (LAR) (6). Extra-levator APR had better circumferential radial margin (CRM) outcome and local recurrence rates than conventional APR (7). 

Güven and Aksel concluded that in the era of routinely used neo-Adjuvant Chemo radiation therapy (NACRT), extra-levator APR is not better than conventional APR for stages 2 and 3. Extra-levator APR showed more morbidity and had better short-term advantage than APR (8, 9). Intersphincter resection is a safe alternative to extralevator APR (10). Malone et al. developed an appendicostomy method to improve the postoperative defecation function in cases with hereditary anorectal deformities, and this method was found to be pseudo-fecal control in 75% of patients (11). Patients with perineal colostomy require retrograde colon enema to regulate lifelong fecal drainage (12). It has been shown to be satisfactory in terms of the Qol and fecal control in patients with perineal colostomy and use of MACE (13, 14).Some authors acknowledged that the combination of Pseudo Continent Perineal Colostomy (PPC) and appendicostomy had provided an acceptable fecal control and improved functional and emotional Qol in cases that had undergone APR (13). Low anterior resection syndrome (LARS) included fecal incontinence or urgency, frequent or fragmented bowel movements, difficulty in rectal emptying, and excessive intestinal gas that occur after a sphincter-sparing rectal resection. MACE is a way to improve the gas and fecal control in patients who have undergone an ISR. Therefore, this combination method can be considered as an alternative to permanent colostomy in carefully chosen cases. This study evaluated Qol in low rectal cancer cases who had undergone ISR with and without MACE, to help reduce the long-term negative outcomes of these patients. The study hypothesis is that ISR with MACE provides an acceptable QoL improvement in ultra-low rectal cancer patients. 

## Methods

**Patients: **This randomized, parallel, prospective single-blind, clinical study was done in 2 years from December 2016 to February 2018 in Sari Imam Khomeini Hospital. The Medical Ethics Committee of MAZUMS (Reference number: IR.MAZUMS.REC.95.2350) approved the study protocol. Before the study, all of the participants signed a written informed consent. The Iranian Registry of Clinical Trials was used to register this clinical trial. (IRCT20141218020364N9). The study's inclusion criteria were all patients with stage 1 (T1-2, N0) of types 2 and 3 of Rullier's classification of low rectal tumor, stage 2a (T3, N0), those who received neo-adjuvant chemo radiotherapy of types 2 and 3 of Rullier's classification of low rectal cancer as confirmed by an endo-sonography survey and pathologist faculty member based on the World Health Organization criteria.

The following were the exclusion criteria: 1) Chronic pulmonary disease; 2) Heart failure and 3) Diabetes; 4) Mental retardation; 5) High dose corticosteroid therapy; 6) Immune deficiency; 7) Poor follow up condition; 8) and other diseases that have an effect on wound healing. A total of 50 patients were found to have stages 1 and 2 of rectal cancer (received neo-adjuvant chemo-radiotherapy courses) and were ready for an open intersphincter rectal cancer resection. Low rectal cancer was identified as tumor less than six centimeters from the anal verge. Types 2 and 3 of Rullier's classification were included (15). Endorectal ultrasound and/or pelvic magnetic resonance imaging (MRI) were used in the staging process.

Rullier's classification includes four subtypes: type I (supra-anal tumors: >1 cm from anal ring), type II (juxta-anal tumors: less than 1 cm from anal ring), type III (intra-anal tumors: internal anal sphincter invasion), and type IV (transanal tumors: external anal sphincter invasion). Sphincter-preserving surgery was possible in 79 percent of patients with low rectal cancer after classification and standardization of surgery based on Rullier's classification (15).

**Figure 1:**
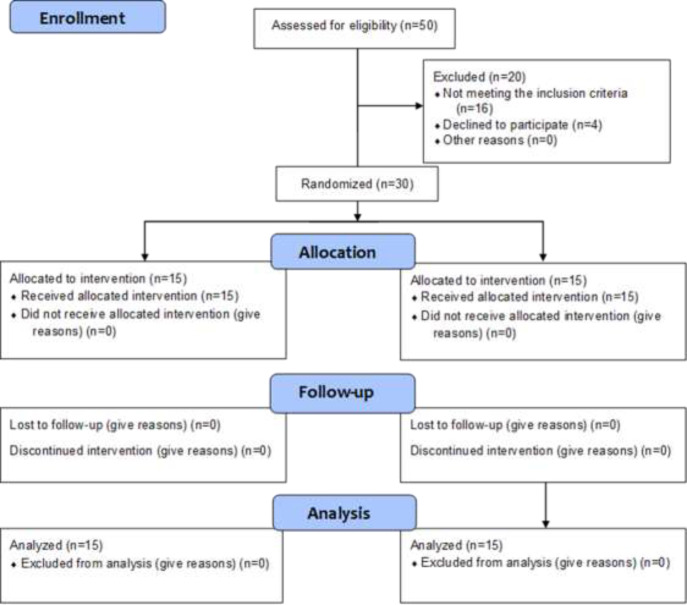
The CONSORT diagram showing the allocation process throughout the trial

16 patients who did not meet the exclusion criteria and 4 patients who refused to participate in the study were removed from the study, and 30 patients were eventually recruited (figure 1). Patients were educated on the clinical aspects of surgical procedures, and written informed consent was obtained from all individual participants in the study; they were then referred to a colorectal clinic for randomization. Participants in this single-blind study were familiar with two forms of rectal cancer surgery. Furthermore, the observer who filled out the questionnaire was unfamiliar with the groups. Similarly, the data analyst was unaware of the study groups. However, the main researcher (surgeon) was made aware of the groups, and both surgical procedures were carried out by the same surgeon. 

**Allocation process: **Patients were assigned into two groups, i.e., Intersphincteric resection with MACE (group 1; 15 patients) and Intersphincteric resection without MACE (group 2; 15 patients), by randomly drawing sealed envelopes, using a computer-based table of randomization.

**Intervention and follow-up: **Surgery was performed under general anesthesia in the lithotomy position for all patients. The patients were administered intravenously with 1 gram of cefazolin (Exir, Iran) as prophylaxis antibiotic on the operating table. Both operating sites were cleaned with 10% povidone iodine solution. 

Surgery was performed in group 1 as two steps. First, after the release of the descending colon, the inferior mesenteric artery and vein were highly ligated. The sigmoid colon was removed from the middle portion. Then, the perineal phase was done and via intersphincter groove complex, the internal anal sphincter, the rectum and distal sigmoid were resected and then end to end colo-anal anastomosis using polyglactin 3.0 suture (Ethicon US, LLC, Cincinnati, Ohio, USA) was performed. At the same time, an appendicostomy was created for the patient, as defined by Azizi et al. (11). The appendix was dissected and the longitudinal incision of the distal appendices was done; then, in the right lower quadrant on the skin a U shape incision was made and the anti-mesenteric edge was sutured to the skin with a 4-0 absorbable suture (Ethicon US, LLC, Cincinnati, Ohio, USA). A small catheter was placed in the appendicostomy for a week and the first antegrade continence enema with one liter of water was done on the table. The fascia layer was closed using nylon 1 (Ethicon US, LLC, Cincinnati, Ohio, USA), and the skin was closed with a 3/0 polypropylene mattress suture (Ethicon US, LLC). The necessary care and method of using appendicostomy was given to patients before discharge. The procedure in group 2 was completely the same in group 1 without creating appendicostomy.

The patients were evaluated for the incidence of abdominal and perineal surgical site infections and hospitalization time during post-op admission. After discharge, the EORTC QLQ-C30 score questionnaire, adjusted and approved by the European Organization for Research and Treatment of Cancer Quality of Life, was monitored for quality of life at 3-, 6- and 12-months’ post-surgery. The WEXNER standard questionnaire was also used to evaluate the defecation control and functional outcomes. Quality of life score of EORTC-QLQ-C30 and evaluation of continence and anal function using Wexner questionnaire (time frame: 1 year) at pre-operative day and 3, 6 and 12 months after surgery were the primary outcomes of the study. LARS score was measured using LARS score in the adult questionnaire. This score scale includes 5 sub-topics: Incontinence for flatus, Incontinence for liquid stool, fecal frequency, clustering of (less than one hour between) bowel movements and Urgency. Zero to 29 points and 30 to 42 points were considered as minor and major LARS, respectively.

Data collection was done by a surgical assistant using the Quality-of-Life Measurement Questionnaire. The Persian translated EORTC QLQ-C30 questionnaire had 30 questions and was specifically designed to assess the quality of life in patients with cancer. Its reliability and validity have already been confirmed in previous studies. This quality-of-life questionnaire was compiled by 15 multivariate scales including 5 functional scales (physical, role, cognitive, emotional and social), 9 signs and symptoms scale (fatigue, pain, nausea and vomiting, dyspnea, sleep disturbances, loss of appetite, constipation, diarrhea and the economic effects of the disease). Scoring was done using the EORTC Designer's Guide, according to which the scores obtained in all areas are 0-100. To determine the quality of life, the data were classified into three good grades (over 75), moderate (50-75) and poor (less than 50) after analysis.

Mean EORTC QLQ-C30 questionnaire value of 3 scores (3, 6 and 12-month post-surgery) was used to compare the groups. The Cleveland Clinica Florida (Wexner) fecal incontinence questionnaire was also used to evaluate the continence control and functional outcomes, and each variable was scored (16).

**Statistical Analysis: **SPSS Version 22, SPSS Inc., was used to record and analyze the collected data. A p-value of less than 0.05 was regarded as statistically significant. The variables were summarized using descriptive statistics such as mean, standard deviation, and frequency tables for quantitative data variables and frequency tables for qualitative data variables. The data is then analyzed using analytic statistical methods such as chi-square and regression methods and generalization equations.

## Results

Twenty of the fifty patients who enrolled in the study were excluded. As a result, the total number of patients included in the study was 30, with 12 females (40 percent) and 18 males (60 percent). Four patients were unable to participate in the study, and sixteen were excluded due to diabetes, chronic pulmonary disease, heart failure, high dose corticosteroid therapy, and poor follow-up.

The final analysis included 15 patients from each group. Figure 1 shows the CONSORT flow diagram for the patients. Demographic data, the cost of surgery ($), hospital stay (day), wound infections, histopathology data and LARS score are mentioned in table 1. The ISR group had similar hospital stay (P=0.106) and lower costs, compared with the ISR+ MACE group (p <.001). No patient had protective stoma. The patients were in stages 1 and 2a and had sufficient margin for safe colorectal anastomosis. Based on two colorectal surgeons’ evaluation and decision, there was no need for protective ileostomy. All patients had uneventful post-op period such as anastomosis leakage. There was no suspected CRM positive patient and all patients were R0. The mean ± SD of the age of the patients in each group were 58±7.45 and 55.53±11, respectively (P=0.43). 

All participants had a median age of 60 years; the mean BMI was 29.26 kg/m2 and 24 (80%) patients were categorized as American Society of Anesthesiologists (ASA) class I–II. Twelve (40%) patients were in stage 1. Eighteen (60 %) patients were in stage 2a (T3, N0) and received complete neo-adjuvant chemo-radiation treatment. 

All pathology reports were well differentiated as adenocarcinoma of rectal cancer. The median tumor distance from the anal verge on table digital rectal examination was 4 cm (range 2–5.5 cm).

**Figure 2:**
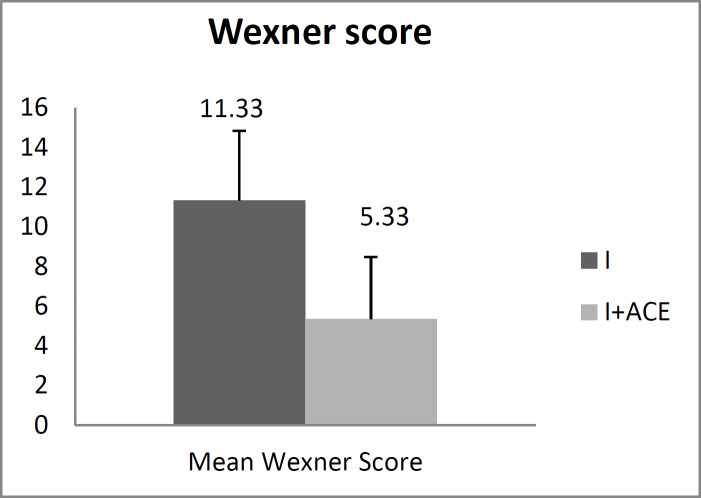
Mean Wexner score in ISR and ISR plus MACE groups

**Quality of life: **In the ISR plus MACE and ISR groups, the overall quality of life score was 76.11 12.55 and 65.56 16.63, respectively. In terms of overall quality of life, there was a significant difference between the two groups (P=0.023). The QoL scores ranged from 0 to 100, with higher scores indicating better status. The overall quality of life was lower in women than in men in both groups (women, 56.24± 13.35 vs. men, 79.56±14.63, P=0.013)**. **The baseline overall Qol score was less than post-operative Qol scores in both groups. (P=0.0001)

**LARS Score: **The ISR plus MACE group had a lower LARS score than the ISR group (25.27 vs. 30.13 4.78; P= 0.030). Major LARS occurred in 53.33 percent (8/15) of ISR group patients and 26.66 percent (4/15) of ISR plus MACE group patients (table 1).

**Table 1:** Descriptive, histopathologic, intra- and post- operative data of study groups

**P-Value**	**ISR plus MACE (N=15, %) ** **(Mean±SD)**	I**SR (N=15 ,%) ** **(Mean±SD)**		
.430	58±7.45	55.53±11	Age	
0.99	9(60)	9(60)	Male	Sex
	6(40)	6(40)	Female	
0.202	29.93±2.46	28.6±2.44	BMI (wt/m^2^)	
0.539	3.6±1.15	3.9±1.03	**Tumor distance from the anal verge** (cm)	
<0.0001	217.33±18.21	178.66±18.65	**Duration of surgery (min)**	
0.106	6.93±1.53	6.2± 1.32	Hospital stay (day)	
0.002	$4,244.27±$728.20	$3,370.47±$616.22	The cost of surgery ($)	
0.030	25±7.27	30.13±4.78	LARS score	
	2(13.33)	1(6.66)	Wound infection	
0.361	11(73.33)	13(86.66)	I–II	ASA class
	4(26.66)	2(13.33)	III-IV	
0.99	15(100)	15 (100)	Well differentiated adenocarcinoma	Histology
0.754	4(26.66)	3(20)	T1(Tumor invade Submucosa)	Tumor Stage (T)
	3(20)	2(13.33)	T2( Tumor invade muscularis properia)	
	8(53.33)	10(66.66)	T3( Tumor invade through muscularis properia into subserosa or into nonperitonealized pericolic or perirectal tissues)	
0.456	7(46.66)	5(33.33)	Stage 1	TNM Stage
	8(53.33)	10(66.66)	Stage 2a	
0.456	8(53.33)	10(66.66)	NACRT	

ASA: American Society of Anesthesiologists; NACRT: Neo-adjuvant Chemo radiotherapy; BMI: Body Mass Index; LARS: Low Anterior Resection Syndrome.

**Wexner Scale Questionnaire: **The overall defecation control and incontinence status score revealed better scores in the ISR plus MACE group than the ISR alone group (5.33±3.13 vs. 11.33±3.50, p<0.0001). The Wexner score ranged between zero and 20 and lower scores meant better status (figure 2).

**Figure 3:**
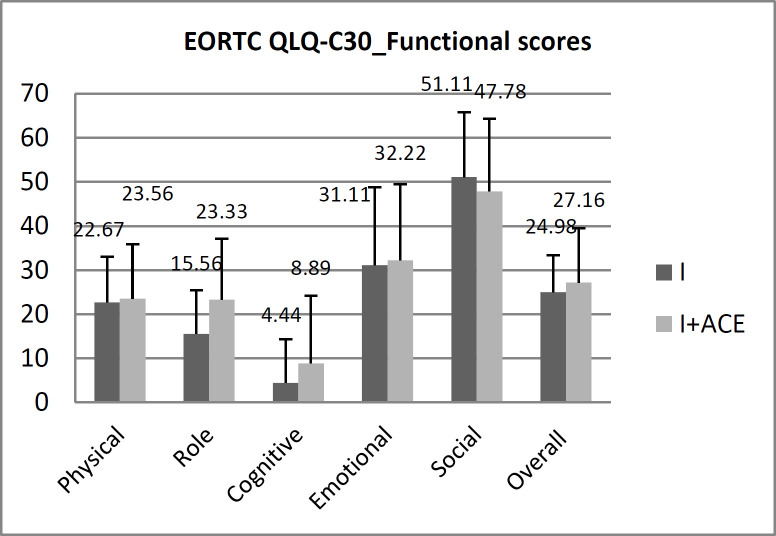
Mean EORTC QLQ-C30_Functional score in ISR and ISR plus MACE groups

**EORTC QLQ-C30 Questionnaire:** Table 2 summarizes the overall scores for QoL functions, and problems included in EORTC QLQ-C30. The mean score for the overall functional QoL experienced by patients who had undergone ISR and ISR plus MACE was 27.1612.34 and 24.98 8.40, respectively, in the “functional” section. (P=0.852) In both groups, the highest functional component was social, while the lowest was cognitive (figure 3). 

**Figure 4:**
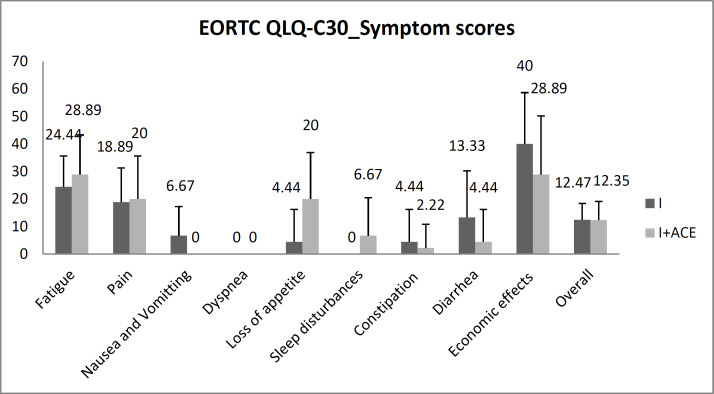
Mean EORTC QLQ-C30_symptoms score in ISR and ISR plus MACE groups

The mean score for the overall problem QoL experienced by patients who had undergone ISR alone and ISR plus MACE was 12.47±5.90 and 12.35± 6.84, respectively in the problem section. (P=0.868). The highest scores in the questionnaire's problem section belonged to those for economic effects and fatigue in both groups (figure 4). The functional component and problem domain scores ranged from 0 to 100, with higher scores representing worse problems and lower scores indicating better index status. The existence of the problem in the social and emotional domain in both groups was more pronounced and the cognitive domain was in the best condition.

**Table 2:** Comparison of functional and sign and symptoms scales between two groups

**P-Value**	**ISR plus MACE (%)** **Mean±SD**		I**SR (%) ** **Mean±SD**		**Group**	
0.949	23.56±12.31		22.67±10.33		Physical	Functional Scales
0.087	23.33±13.80		15.56±9.89		Role	
0.518	8.89±15.26		4.44±9.89		Cognitive	
1.000	32.22±17.21		31.11±17.6		Emotional	
0.511	47.78±16.51		51.11±14.73		Social	
0.852	27.16±12.34		24.98±8.40		Overall	
0.320	28.89±14.43		24.44±11.27		Fatigue	Signs and symptoms scale
0.878	20.00±15.69		18.89±12.39		Pain	
0.016	0.00±0.00		6.67±10.54		Nausea and Vomiting	
1.000	0.00±0.00		0.00±0.00		Dyspnea	
0.029	20.00±16.90		4.44±11.73		Loss of appetite	
0.073	6.67±13.80		0.00±0.00		Sleep disturbances	
0.550	2.22±8.61		4.44±11.73		Constipation	
0.104	4.44±11.73		13.33±16.90		Diarrhea	
0.140	28.89±21.33		40.00±18.69		Economic effects	
0.868	12.35±6.84		12.47±5.90		Overall	
P-Value (ISR/ISR+MACE)	ISR+MACE	Base line score	ISR	Base line score	Overall Quality of Life Score (Good >75, Moderate 50-75, Weak<50)	
0.023	76.11±12.55	39.06±9.53	65.56±16.63	42.66±9.3		
	0.0001		0.0001		P-Value (Baseline/ ISR; Baseline/ ISR=MACE)	

ISR: Intersphincteric Resection, MACE: Malone antegrade continent enema

## Discussion

Quality of life has been identified as one of the most important clinical research topics, as well as one of the most effective aspects of cancer patient care, and its assessment has been used to detect differences between patients, predict disease outcomes, and evaluate therapeutic interventions. Cancer causes a disordered occupation, economic status, social status, family life and the destruction of the patient's life including mental, psychological, social, economic, and sexual function. The purpose of this study was to compare the quality of life and functional outcomes of patients with early-stage low rectal cancer who had undergone ISR plus MACE versus ISR alone using the EORTC-QLQ-C30 and Wexner questionnaires at 3, 6, and 12 months after surgery, as well as the LARS score at 12 months after surgery.

Wexner score improved in the ISR plus MACE versus ISR alone (5.33 vs. 11.33). The overall quality of life score was better in the ISR plus MACE versus ISR alone and the baseline scores in both groups. Our finding also indicated that the overall quality of life was lower in women than in men in both groups. The results of the present study showed that patients who had undergone ISR alone had lower scores in terms of physical, role, emotional and cognitive aspects and had a worse score in social status. In our study, the emotional score increased to 32.22 in patients who had undergone ISR plus MACE compared with those without MACE (31.11), and the cognitive score rose to 8.89. The social and emotional problems had higher scores in both groups and the cognitive dimension was in the best condition. In our study, the social dimension improved in the ISR plus MACE patients versus ISR alone patients (51.1 vs. 47.78, P>0.05) 

In the Dumont’s study(17), quality of life and continence were comparable in the ISR and APR plus perineal colostomy groups for ultralow rectal cancer. Patients who had only undergone ISR had more defecation issues and evacuation difficulties. In Konanz et al.’s study (6), the APR method performed worse than the ISR. Physical functioning improved significantly after sphincter preservation surgery compared to APR. Wexner scores after ISR were significantly higher (12.9) than after LAR (9.5).

In the Azizi et al.’s study, based on the EORTC QLA-C30 questionnaire, overall QoL and Wexner scores were 78 and 7 in the PPC plus appendicostomy patients, respectively. Our results are consistent with those of Parker et al.’s study which was performed on 351 cancer patients using the SF-12 questionnaire and showing lower Qol score in women (16). Schultz’s study was conducted on 344 rectal cancer patients using the FACT-G questionnaire in the United States, showing significantly higher quality of life scores in women (18). These differences may be due to the differences in the quality-of-life measurements, the research community and the number of samples examined. We believe that women have a central role in the family. Their greater responsibility towards the family and the caring their children cannot take over this responsibility following disease condition and the long stages of treatment, which causes tensions and psychological stresses in them. 

Recent studies have shown a decline in the functional status with quality of life associated with health in colorectal cancer patients although this relationship may be weaker in long-term survivors of cancer(19). A recent study has shown lower pre-operative functional status with lower physical scores using QLQ-C30/CR38 and CF-12 questionnaires (20, 21). Based on the EORTC QLQ-C30 questionnaire, role, cognitive, and functional status in Azizi et al.’s study (13) received the highest scores (100, 100, and 93) in association with appendicostomy.

In the present study, LARS score was lower in the ISR plus MACE group. Major LARS was reported in half of the subjects in the ISR group and one third of ISR plus MACE patients. It seems that colonic irrigation using MACE could decrease the LARS symptoms in patients who had undergone LAR using ISR procedure. LARS was very commonly reported after low anterior rectal resection and improved during 2 postoperative years, but it persisted longer in nearly 60% of patients, 50% of whom had the major form. A minority of cases had access to several therapeutic strategies available such as motility drugs colonic irrigations (e.g. MACE), and sacral neuro-modulation (22). Sacral nerve stimulation (SNS) in adults with fecal incontinence who had not responded to medical therapy resulted in > 50% improvement in symptoms in approximately 80% of patients. According to recent reviews, SNS for fecal incontinence in LARS has had success rates comparable to its use for other types of fecal incontinence (23, 24). Further surgery and hospitalization cost was reported in the ISR + MACE group patients in our study. Since MACE procedure increases the costs and surgery duration to be done in all patients with low rectal cancer, it is recommended that this procedure should be performed on demand when incontinence and LARS are not treated otherwise. The strengths of our study are the prospective data collection, the use of a validated questionnaire, and the high rate of questionnaire completion by the patients. However, there are also limitations. First, the sample size of patients was relatively small. Second, our 1-year follow-up period was relatively short. Third, baseline overall QoL score was added to the study, retrospectively. Fourth, no objective measurement of bowel function such as anorectal manometry was performed. As the information available about MACE for patients with early-stage rectal cancer treated with MACE at the time of the study design was zero, no power calculation was performed for these outcomes. Despite these limitations, the results of this trial should be used in future studies comparing the outcomes of ISR and MACE with other treatment modalities. In conclusion, in our prospective trial of patients with early stage (T1-2N0, T3N0) low rectal cancer treated with ISR plus MACE, the functional score was similar to the ISR alone patients one year after the surgery. 

However, during the same postoperative period, patients reported a significant decrease in nausea and vomiting domains and LARS score of the ISR plus MACE patients. Overall, QoL in ISR plus MACE patients was better than those without MACE. Female sex may have an impact on QOL outcome. Given all the above, it is best to perform an appendicostomy on demand in the second procedure via McBurney's incision when incontinence and LARS are not treated otherwise.
